# Editorial: The gut-kidney axis: A potential drug target for treating kidney diseases

**DOI:** 10.3389/fphar.2022.1012890

**Published:** 2022-09-07

**Authors:** Jianping Chen, Karl W. K. Tsim, Kun Gao, Mahyar Khazaeli

**Affiliations:** ^1^ Shenzhen Key Laboratory of Hospital Chinese Medicine Preparation, Shenzhen Traditional Chinese Medicine Hospital, The Fourth Clinical Medical College of Guangzhou University of Chinese Medicine, Shenzhen, China; ^2^ Division of Life Science and Center for Chinese Medicine, The Hong Kong University of Science and Technology, Hong Kong, China; ^3^ Division of Nephrology, Affiliated Hospital of Nanjing University of Chinese Medicine, Nanjing, China; ^4^ Department of Pathology, University at Buffalo, Buffalo, NY, United States

**Keywords:** gut-kidney axis, kidney disease, gut microbiota, microbial metabolism, therapeutic strategies

Gut microbiota, comprising trillions of microorganisms, have been proved to have significant impact on human health and disease. The microbiota play essential roles in the function and development of several physiological processes, including metabolism in kidney (Fan and Pedersen, 2021). Recent studies have shown the bidirectional interactions within the gut-kidney axis. The kidney diseases could be caused by dysbiosis of gut microbiota, while the imbalance of microbial metabolism directly contributes to the altered population of gut microbiota that could cause the progression of kidney diseases (Hobby et al., 2019). Preclinical and clinical observations implicate that the dysfunction of gut-kidney communication could be identified during the pathogenesis of chronic kidney disease (CKD), diabetic nephropathy, hypertension nephropathy, and acute kidney injury (AKI). However, the detailed information of these molecular mechanisms along gut-kidney axis in kidney disorders is still very limited. The goal of this research topic is to provide a forum for advance research on gut microbiome and its metabolites in regulating gut-kidney axis during the progression of diseases.

The review by Huang et al. summarized the findings of gut microbiota in progression of CKD and CKD-associated complications, i.e., cardiovascular disease (CVD). Gut microbiota have beneficial effects on the host, e.g., maintaining integrity and function of gastrointestinal tract, as well as strengthening immunological effects. The dysbiosis of microbiota in gut contributes to the development of CKD and CVD. The metabolites, or the pro-toxins originated from microbial metabolism under renal dysfunction, can be subsequently metabolized by the host to generate toxins, i.e., p-cresyl sulfate and indoxyl sulfate: these toxins damage the cardiovascular system and accelerate progression of CKD. The authors proposed the concept of “gut-kidney-heart” axis. The idea suggests a close interaction of gut, kidney and heart, and the dysfunction of this interplay leads to CKD progression and complication. Therefore, the modulation of gut-kidney-heart axis could be promising therapeutic strategies for CKD and CVD; these strategies included dietary intervention, probiotics, prebiotics, genetically engineered bacteria, fecal microbiota transplantation, bacterial metabolite modulation, antibiotics, and traditional Chinese medicine.

In order to understand the gut-kidney axis in anti-neutrophil cytoplasmic antibody (ANCA)-associated vasculitis (AAV), Yu et al. conducted a cross-sectional collection of fecal samples from 23 AAV patients having kidney injury, and which was subjected to analyses of gut microbial composition, taxonomic difference, and prediction of microbial function by using 16S rRNA microbial profiling approach. The authors have found that the richness and diversity of gut microbiota could be reduced in AAV patients having kidney injury. Furthermore, Spearman’s correlation analysis was employed to determine the correlation between gut microbiota and the observed clinical indicators, including serum creatinine (SCR), blood urea nitrogen (BUN), estimated glomerular filtration rate (eGFR). The results indicated that alteration of gut microbiome, especially Deltaproteobacteria, Bacteroidales, Desulfovibrionaceae, Paraprevotella, and Lachnospiraceae groups might be associated with the severity of kidney injury of AAV patients. For instance, the abundance of Lachnospiraceae group was lower in AAV patients having kidney injury than the healthy control, which was negatively correlated with SCR and positively correlated with eGFR.

Rhubarb is the root and rhizome of *Rheum palmatum*, which is being used as a Chinese herbal medicine and as an ornamental plant in garden. Li et al. investigated the effect of rhubarb on CKD rats through the regulation of intestinal microbial community. Trimethylamine-N-oxide (TMAO) is an end-product of choline and carnitine metabolism. Under metabolism of gut microbiota, choline and carnitine can be converted to TMA, and then which is oxidized to TMAO in host’s liver. TMAO is considered as a biomarker of renal dysfunction. The classic 5/6 nephrectomy (Nx) CKD rat model was used in this study, and the TMAO-targeted metabolomics was measured by UPLC-MS/MS analysis. In this article, the authors have found that the enema treatment of rhubarb extract significantly reduced circulating TMAO level, and parallelly the regulation of intestinal microbial community. In parallel, Spearman’s correlation analysis revealed that Lachnospiraceae and Romboutsia were positively correlated with the level of TMAO. Hence, rhubarb enema decreases circulating TMAO level and alleviates renal fibrosis in 5/6Nx CKD rats, which may be involved in regulation of gut microbiota.

Two articles focusing on different aspects of therapeutic targets in treating kidney diseases have been included in this special issue. Cui et al. determined the roles of SET and MYND domain protein 2 (SMYD2) in AKI. The authors have found that SMYD2 was highly expressed in a murine model of cisplatin-induced AKI. The blockade of SMYD2 by using specific siRNA suppressed the cisplatin-induced apoptosis of cultured HK-2 cells. Another study by Ren et al. demonstrated the effects of microcystin in stopping progression of kidney fibrosis. The operation of unilateral ureteral obstruction (UUO) was conducted to induce renal fibrosis in mice. The authors have found that microcystin protected the UUO-induced renal fibrosis, and the mechanism of which was proposed having the inhibition of pyruvate kinase M2 (PKM2)-hypoxia inducible factor (HIF)-1α.

In summary, this research topic has discussed the critical roles of gut microbiota and its metabolites in progression of kidney diseases, as well as possible therapeutics in targeting the gut-kidney axis. These studies enrich our understanding in contribution of gut-kidney axis in progression of kidney diseases, which can be helpful to develop novel therapeutic approaches and future research directions.
